# Spatio-temporal brain dynamics of self-identity: an EEG source analysis of the current and past self

**DOI:** 10.1007/s00429-022-02515-9

**Published:** 2022-06-07

**Authors:** Francisco Muñoz, Miguel Rubianes, Laura Jiménez-Ortega, Sabela Fondevila, David Hernández-Gutiérrez, José Sánchez-García, Óscar Martínez-de-Quel, Pilar Casado, Manuel Martín-Loeches

**Affiliations:** 1 Center UCM-ISCIII for Human Evolution and Behavior, Avd. Monforte de Lemos, nº 5, Pabellón 14, 28029 Madrid, Spain; 2 Psychobiology and Methods for the Behavioral Sciences Department, Complutense University of Madrid, Madrid, Spain

**Keywords:** Source localization, Self-identity, Self-continuity, Cluster-based permutation test

## Abstract

Current research on self-identity suggests that the self is settled in a unique mental representation updated across the lifespan in autobiographical memory. Spatio-temporal brain dynamics of these cognitive processes are poorly understood. ERP studies revealed early (N170-N250) and late (P3-LPC) waveforms modulations tracking the temporal processing of global face configuration, familiarity processes, and access to autobiographical contents. Neuroimaging studies revealed that such processes encompass face-specific regions of the occipitotemporal cortex, and medial cortical regions tracing the self-identity into autobiographical memory across the life span. The present study combined both approaches, analyzing brain source power using a data-driven, beamforming approach. Face recognition was used in two separate tasks: identity (self, close friend and unknown) and life stages (childhood, adolescence, adulthood) recognition. The main areas observed were specific-face areas (fusiform area), autobiographical memory areas (medial prefrontal cortex, parahippocampus, posterior cingulate cortex/precuneus), along with executive areas (dorsolateral prefrontal and anterior temporal cortices). The cluster-permutation test yielded no significant early effects (150–200 ms). However, during the 250–300 ms time window, the precuneus and the fusiform cortices exhibited larger activation to familiar compared to unknown faces, regardless of life stages. Subsequently (300–600 ms), the medial prefrontal cortex discriminates between self-identity vs. close-familiar and unknown. Moreover, significant effects were found in the cluster-permutation test specifically on self-identity discriminating between adulthood from adolescence and childhood. These findings suggest that recognizing self-identity from other facial identities (diachronic self) comprises the temporal coordination of anterior and posterior areas. While mPFC maintained an updated representation of self-identity (diachronic self) based on actual rewarding value, the dlPFC, FG, MTG, paraHC, PCC was sensitive to different life stages of self-identity (synchronic self) during the access to autobiographical memory.

**Supplementary Information:**

The online version contains supplementary material available at 10.1007/s00429-022-02515-9.

## Introduction

When thinking or looking at photographs, people bring familiar episodes of themselves and their social relatives back from the past (e.g., childhood, adolescence). At the psychological level, albeit moving along discrete moments in the autobiographical scale (synchronic self, Carruthers, 2006; Doering et al. 2012), the core self keeps constant as we recognize ourselves vs. others regardless of time passing (diachronic self; Slors 2001; Northoff 2017). Moreover, as social selves, personal identity develops from childhood to present with other selves (Gilliham and Farah 2005). At this point, a hypothetical function of the self may be to discriminate between self and other-selves as a critical role in psychological development, based on physical, memory, rewarding, and affective values (Janczyk et al. 2019; Mascolo 2019; Sui and Humphreys 2015). At the neurocognitive level, disentangling brain dynamics (i.e., when and where in the brain) of stability and fluidity of mental representations may offer empirical insights into how the brain processes support the social self and how it evolves across time.

### The face as the critical representation of the evolving identities

A face ensues the unique physical representation of the self (Tsakiris 2017), and self-portraits are reliable markers of self-awareness (Butler et al. 2013). It is largely demonstrated that one’s face prompts prioritized access to cognitive resources compared to other faces (Alzueta et al. 2019; Sui and Rotshtein 2019). In this access, a coarse-to-fine course may be followed (Dobs et al. 2019). A first step in processing the global facial configuration is substantiated by face-specific areas identifying *who this person is* (Kanwisher and Yovel 2006; Haxby et al. 2000; Weiner and Grill-Spector 2012). The face fusiform gyrus (FG) is core in mediating identity recognition based on structural features irrespective of familiarity (Kanwisher and Yovel 2006; Shah et al. 2001). Previous evidence points to the temporoparietal junction (TPJ) to be also relevant in self-face representation (Apps et al. 2012, 2015; Platek et al. 2006; Tsakiris et al. 2008; Uddin et al. 2006). Interestingly, parietal areas seem to track changes in the self across time. As an example, Uddin et al. (2006) observed that the right inferior parietal cortex might also be involved in integrating both past and current self-facial configuration. Apps et al. (2012, 2015) have found that a set of unimodal and multimodal areas are sensitive to self-adulthood identification. It has been shown that fronto-parietal attentional networks could be recruited by the presence of self-related stimuli (Humphreys and Sui 2016; Zhao et al. 2018). Hence, the role of the inferior parietal areas deserves more dedicated studies to clarify whether it entails a static representation of the physical identity.

In a second step, a set of distributed areas enables full recognition of the personal identity after accessing accrued information in long-term memory (relevant person-identity information in the lifespan), offering valuable insight into *how this person is* (Gobbini and Haxby 2007; Tanguay et al. 2018; Góngora et al. 2019). Neuroimaging studies have described that posterior cortical regions (mainly posterior cingulate cortex -PCC- and precuneus -PC) are crucial for tracing the self into autobiographical memory (Burgess et al. 2001; Gobbini et al. 2004, 2007; Ishai et al. 2000; Sugiura et al. 2005). Additionally, Bobes et al. (2013) observed that anterior regions (e.g., medial prefrontal cortex—mPFC) exhibited stronger and prolonged activity to personal-familiar faces, being weaker to unknown or visually familiar (learned after repetitive exposition) faces. In a self vs. other identity discrimination, Murray et al. (2015) described mPFC activation to be specific to self-identity, motivationally oriented recognition, while PCC/PC would be specific to other-identity socially oriented recognition. In addition, the TPJ and the anterior and medial temporal gyri (ATG, MTG) are involved in taking the perspective of others, suggesting self vs. other discrimination and self-knowledge processing (Knyazev et al. 2018; Sheldom et al. 2019; Sugiura et al. 2006). Interestingly, anterior cortical regions (particularly ventromedial prefrontal cortex -vmPFC- and anterior cingulate cortex -ACC) dissociated current and past representations of self-referential information (D’Argembeau et al. 2008, 2010; Northoff 2017). The question here is how anterior and posterior parts are coupled throughout time course when recognizing the same face identity at different life stages. To our knowledge, this point is not well understood so far.

Previous literature has observed that face-specific areas and autobiographical-specific areas are jointly activated to self-identity recognition processing as a whole (Sugiura et al. 2008, 2012; Apps et al. 2015). The *identity-value model* (Berkman et al. 2017) intends to unify face-identity and autobiographical content in salience, reward value, and affect-based motivational concept. Self and personal-familiar stimuli, including faces, are featured by their salience and emotional content (Damasio et al. 2000; Gobbini et al. 2004; Maddock 1999). Several studies observed that self-faces and close-friend faces facilitate their recognition processing, compared to unknown faces, based on personal knowledge attached to each identity (Alzueta et al. 2019; Apps et al. 2012; Kotleswka and Novicka 2015; Rubianes et al. 2020; Woźniak et al. 2018). Specifically, Ramon and Gobbini (2017) pointed to the optimization of visual (detection and identification) and non-visual (person knowledge and emotional responses) processes when distinguishing familiar and unfamiliar faces.

Nonetheless, self-face processing is boosted the most as additional processes are involved (memory, reward, and emotions). Attending to the temporal scale of autobiographical memory, face-specific areas would feed those brain areas of the Cortical Midline Structure (CMS, Northoff 2017; Northoff and Bermpohl 2004), for instance, vm/dmPFC, ACC, PCC, PC that entails the projection of personally familiar identities to past, present, and future moments encoded in autobiographical/episodic memory. In this sense, some studies have found different neural networks to process the current self and past self (Apps et al. 2012; Butler et al. 2013). More precisely, in an fMRI study using morphing self-face with familiar-other face, Apps et al. (2012) observed that activity of face-specific areas (inferior temporal areas) was modulated depending on the amount of resemblance to the current self of morphed images. Moreover, they found that memory-specific areas (hippocampus -HC- and PCC) and areas related to the sense of body ownership (temporo-parietal junction and inferior parietal) were modulated depending on self-content in the past. While it is expected that the rewarding value attached to identity discriminates between self-identity and other identities (unknown and close-familiar) (Northoff and Hayes 2011), it is not clear that such value attached to the self would decrease from present to past. If this is the case, such a value should be greater to the self than to close-familiar and unknown identities. The relationship between past self compared to past other in terms of rewarding value is also a matter of intense debate (D’Argembeau et al. 2008, 2010; Butler et al. 2013; Kotlewska and Nowicka 2015; Rubianes et al. 2020).

### Temporal course of neural correlates of the evolving self-identity

The temporal course of brain dynamics relative to face identity processing as evolving in the timespan has been scarcely described. Recent ERP studies have unraveled the temporal course of brain potentials when recognizing the identity of a face (e.g., Alzueta et al. 2019; Kotlewska and Novika 2015; Rubianes et al. 2020; Woźniak et al. 2018). Several ERP studies have systematically found specific components indexing facial configuration processing. The N170 component has been related to structural face processing (Bentin and Deouell, 2000), with minimal sensitivity to familiarity (Eimer 2011). By contrast, the N250 component is associated with familiar face recognition (Schweinberger et al. 2002). Later ERP components like the P3-LPC (for Late Positive Component) index higher-order cognitive processes, such as allocating attentional resources relative to task demands (Azizian and Polich 2007; Polich 2007), or affective and rewarding processing (Cunningham et al. 2005). In our previous work (Rubianes et al. 2020), we observed early face-specific modulations around 170 ms relative to facial-configuration processing when comparing the processing of face pictures at different life stages. The first instance of self-identity discriminability was found around 250 ms, suggestive of the earliest preferential access to specific autobiographical contents in long-term memory based on emotional saliency and rewarding value, in consonance with previous research (e.g., Miyakoshi et al. 2010; Woźniak et al. 2018). Later latencies (300–600 ms) revealed long-lasting P3-LPC modulations encompassing higher-order self-referential processing and personal significance of face stimuli (Gillihan and Farah 2005; Tanguay et al. 2018). In this regard, Kotlewska and Novicka (2015) also observed an enhanced P3 amplitude to present self, compared to close-friend, famous and unknown faces and names, suggesting that this component may be an index of accessing personal semantic knowledge. Such P300 increment was not different between present and past selves. Importantly, LPC modulations were especially sensitive to self vs. other identity distinctions in the temporal perspective of the self. The larger this long-lasting positivity, the more resources allocated to elaborate personal knowledge and self-relevant content (Keyes et al. 2010; Xu et al. 2017). Interestingly, we noticed such self-prioritization processing whether or not attention was oriented to identity (Rubianes et al. 2020). The LPC also revealed self-specificity to life stages, showing higher amplitude to present self compared to a past self, but also dissociating past self from past other. This result was also observed by Kotlewska and Novicka (2015) as an increment of the later positivity amplitude to past self-face compared to close friend face, interpreted as suggestive of different emotional content for self and close-other face related to the past. All these findings evinced the diachronic component (discrimination between self vs. other) but also the synchronic component (discrimination of self at different life stages).

### The present study

The literature reviewed above indicates that recognition of self compared to close-familiar and unknown faces may involve a coordinated activation of different neural networks of specific-face areas (e.g., the fusiform gyrus) and self-reference processing (CMS, e.g., the vm/dmPFC, PC/PCC) and others lateral areas (dlPFC, middle temporal and TPJ) depending on the course of processing (perception, memory, attention, taking the perspective of others, emotion and rewarding) (Berkman et al. 2017; Knyazev et al. 2018; Sui and Humphreys 2015). Moreover, modulations of the activation in that areas might be influenced by the interactions between identities and life stages (Butler et al. 2013; Kotlewska and Novicka 2015; Rubianes et al. 2020). However, knowledge about the specific spatiotemporal course of cortical activation involved in facial-identity recognition between the self-face and the close-friend face is not well delimited in terms of autobiographical memory. The literature reviewed (Butler et al. 2013; Kotlewska and Novicka 2015; Rubianes et al. 2020) does not delve into which areas participate in accessing self-related knowledge, nor do they explore identity at different life stages (childhood, adolescence, adulthood). This study aimed to disentangle the temporal course of data-driven source activations. We expected to find significant source power in regions of interest (ROIs) to face-specific areas (FG) and autobiographical memory areas (mPFC, PCC/PC, ATL, MTG). Data-driven ROIs estimation was accomplished by analyzing the time-based activity of brain source power using a beamforming approach and cluster-permutation statistical tests (for a recent review, see Westner et al. 2022). This approach computes a voxel-level model representing a given source location while suppressing the contribution from nearer sources (Veen et al. 1997). Time-based source power was measured at the same ERPs time windows used in Rubianes et al. (2020). We hypothesized that: (a) at early latencies, source power would be sensitive to global face configuration involving core face-processing areas (Kanwisher and Yovel 2006; Shah et al. 2001), (b) at mid-latencies, posterior brain sources would discriminate self-identity from close-familiar and unknown (Alzueta et al. 2019; Schweinberger et al. 2002); (c) at later latencies, mPFC together with posterior areas would show an increase of activation to self-identity compared to familiar and unknown identities, in support of the diachronic component of the self (Novicka 2015; Rubianes et al. 2020); (d) anterior and posterior brain sources would show late activity modulations specific to self-identity at different life stages in support of the synchronic component of the self (Bobes et al. 2012; Murray et al. 2015). The participants had to recognize the same set of facial stimuli in two different recognition tasks: (a) identity, (b) life stages.

## Methods

### Participants

The sample employed in this study has been described in detail in Rubianes et al. (2020). Twenty undergraduate and graduate students participated in the study (*M* = 23.85 years, SD = 3.93 years; 10 females). All participants reported normal or corrected-to-normal vision and all were right-handed, mean score of + 86 (range: + 63 to + 100) according to the Edinburgh Handedness Inventory (Oldfield 1971). Before the experiment, the participants gave their informed consent. The study was conducted in accordance with the Declaration of Helsinki of the World Medical Association and approved by the Ethics Committee of the Faculty of Psychology of the Complutense University.

### Material

The material and experimental procedure were the same as reported in Rubianes et al. (2020). Stimuli consisted of a set of digitalized photographs of the faces of the participants and one of his/her close friends. Images showed neutral emotional expression and direct gaze. Two weeks before the experiment, the participants provided nine images per identity. From each participant, we collected three digitalized high-quality photographs for each stage of life (childhood, adolescence, and adulthood) of herself (self-identity condition), and another three of one of their same-sex close friends (close friend condition). For the unknown condition, another three photographs were obtained from the close-friend condition of other participants, thereby each participant had a different unknown condition. To increase variability, we collected three different versions of each image (e.g., three images of self childhood, three of self adolescence, etc.). This represents 27 pictures (3 versions of 3 identities and 3 life stages). To enhance the signal-to-noise ratio, each image was repeated 10 times. The picture order was randomized. All were matched in age and sex. All participants included in the study stated informally a wide range of friendship since childhood (6–9 years old), and that the relationship was kept, with contacts held from several times a year to habitually or daily. All images were edited in Adobe Photoshop^®^ (CS6), applying a black background. The luminance was equated across images as much as possible. The images were resized by adjusting a constant distance in the width of 145 pixels between pupils, thus maintaining the height and width proportions of the face. All stimuli were framed within 450 pixels width and 600 pixels height. At the end of the experiment, all participants confirmed that they did not know the face identity of the unknown face.

### Procedure

The experiment was conducted using Presentation^®^ software (Neurobehavioral Systems, Inc.). Participants were seated in an isolated room approximately 70 cm in front of an LCD screen of 1024 × 768 pixels. A typical trial started with a fixation cross for 500 ms presented centrally on the screen in white with a black background, followed by a blank for 200 ms. A picture appeared thereafter for 1000 ms followed by a blank for 200 ms, and, finally, a response interval of 1000 ms. The response interval was detached from the presentation of the picture to avoid movement artifacts on the ERP. The participants provided their responses by pressing one of three buttons. The sequence of the buttons was counterbalanced between the index, middle and ring fingers of the right hand. The experiment was divided into two tasks: (1) identity recognition task, the participants were asked to press a button to discriminate the identity of the face image (self, close-friend, unknown); (2) life stages recognition task, they were asked to identify the face’s stage of life (adulthood, adolescence, and childhood). The task order was counterbalanced between subjects. Overall, 540 stimuli were presented to the participants (27 photographs × 10 presentations × 2 tasks).

### EEG recordings and analysis

EEG settings have been described in detail in Rubianes et al. (2020). Continuous EEG was registered using 59 scalp electrodes (BrainCap; Brain Products, Gilching, Germany) according to the international 10–20 system. EEG data were recorded with a BrainAmp DC amplifier (Brain Products, Gilching, Germany) at a sampling rate of 250 Hz with a band-pass from 0.01 to 100 Hz. During recording, all scalp electrodes plus the left mastoid were all referenced to the right mastoid, which were re-referenced off-line to the average of the right and left mastoids. The ground electrode was located at Afz. The impedance of all electrodes was kept below 5 kΩ. Eye movements and blinks were monitored using vertical (VEOG) and two horizontal (HEOG) electrodes placed above and below the left eye and on the outer canthus of both eyes, respectively.

EEG data were analyzed with the software Brain Vision Analyzer^®^ and EEGLAB v14.1 (Delorme et al. 2011; http://www.sccn.ucsd.edu/eeglab) as a toolbox of Matlab R2017b (MathWorks, Natick, MA, USA). Raw data were filtered offline with a band-pass of 0.1–40 Hz and then segmented into 1200 ms epochs starting 200 ms before the picture onset. The baseline correction was applied from − 200 to 0 ms. Both incorrect and omitted responses were excluded from the analyses, as well as trials with transient noise. Typical artifacts (e.g., eye movements or muscle activity) were corrected through infomax independent component analysis (ICA, Bell and Sejnowski 1995). This analysis decomposed individual EEG data into 64 independent components (ICs) each characterized by a fixed scalp map of the spatial projection of the component to each channel, as well as a time course of activation in each trial. From the overall Ics, we applied a semi-automated procedure described by Chaumon, Bishop, and Busch (2015). This procedure assists with statistical criteria to select artifacted ICs due to eye movements, muscle contractions, line noise or electrode misconnections. After identifying the artifacted ICs, they were dropped out from the EEG data from all electrodes.

After artifacts rejection, the mean of segments for each condition was: self (86.55 ± 3.23); close-friend (86.00 ± 4.45); unknown (87.15 ± 2.97); childhood (84.25 ± 6.13); adolescence (81.45 ± 8.21); adulthood (84.00 ± 5.93). Comparing the overall segments between the identity task (259.7) and the life stage task (249.7) revealed a significant difference (*t*_(19)_ = 2.574, *p* < 0.05). Overall, the mean rejection rate of segments of all epochs was 3.82% on the identity task and 7.52% on the life stages task.

### Source analysis

Source analysis was performed using Fieldtrip (Oostenveld et al. 2011), an open-source MATLAB toolbox, on the preprocessed EEG data. To estimate deep brain sources, we used a scalar beamformer approach, namely, a linearly constrained minimum variance (LCMV) spatial filtering (Van Veen et al. 1997). LCMV computes an adaptative spatial filter whose weights are calculated using both the forward field matrix and the inverse of the covariance matrix obtained by the EEG time-domain data. The brain is divided into a regular three-dimensional grid and the source power for each grid or voxel point is computed, reflecting a given source location while simultaneously suppressing the contributions from other sources and noise contained in the data covariance matrix. To generate the forward model, the lead field matrix was computed based on an EEG head model template (Boundary Element Method, BEM) and divided into a 5-mm-spaced grid (source model). Both head model and source model were aligned with the electrode positions (‘standard_1020.elc’) and adjusted to the same coordinate system (based on the Montreal National Institute atlas). Subsequently, the covariance of all conditions was calculated using the Fieldtrip’s function *ft_timelockanalysis* and a common spatial filter was obtained for each time window using *ft_sourceanalysis*. Thus, the source analysis for each condition is calculated by its corresponding covariance and projected through its common filter for each time window. In line with the ERP results from our previous study (Rubianes et al. 2020), we used similar time windows of interest: N170: 150–200 ms; N250: 250–300 ms; P3 and LPC 300–600 ms.

To test the 3D spatial distribution of source power produced by the LCMV method, non-parametric statistics and cluster-based correction tests were performed using *ft_sourcestatistics* (Maris and Oostenveld 2007). The significance probability is computed under the permutation distribution using the Monte-Carlo method. This statistical test was applied to test the difference between experimental conditions, namely, Identity (self vs. friend; self vs. unknown; friend vs. unknown), Life Stage (adulthood vs. adolescence; adulthood vs. childhood; adolescence vs. childhood), and its corresponding interactions. First, each grid point or sample is contrasted by means of a *t*-value; then all the samples are selected cluster candidates whose *t*-value is larger than some threshold (alpha-cluster = 0.016, *t*-values are thresholded at the 98.4-th quantile for one-sided *t*-test; corrected by the number of comparisons). Selected samples are clustered in connected sets based on temporal, spatial adjacency. The permutation distribution is formed by randomly reassigning the conditions across all participants 8000 times; and for each of these permutations, the cluster candidate who had the highest summed values was compared against the permutation distribution. Differences between conditions were considered significant if the *p*-value calculated for the highest largest cluster-level statistic is smaller than the critical alpha-level (0.05). The output of source-level statistics was interpolated onto an anatomical MRI template (based on MNI coordinates) and plotted the maximum activity. To localize the effects on the source level, a posterior statistical threshold mask was applied to the distribution (*p*-corrected = 0.01, *t* > 2.56). The brain mapping was carried out by locating the MNI coordinates corresponding to the peak t-values. The atlases used were both MRIcron (Rorden and Brett 2000) and Neurosynth (Yarkoni et al. 2011).

## Results

### Behavioural results

The percentage of participants’ performance was as follows (mean ± SD): *hits rates* (identity: 99.03% ± 1.25%; life stage: 92.5% ± 6.28%), *misclassifications* (identity 0.87% ± 1.23%; life stage: 7.25% ± 6.31%), *omissions* (identity: 0.09% ± 0.16%; life stages: 0.24% ± 0.28%). Percentages of misclassifications between tasks revealed a significant difference [*t*(19) = 4.713, *p* < 0.001]. As the response interval started 1200 ms after the presentation of the stimulus, reaction times were considered uninformative and, therefore, were not measured.

### Source analysis results

#### Source reconstruction at 150–200 ms time window

The cluster-permutation test did not yield any significant difference between conditions during this time window. For a detailed description of *p *values of the clusters, see Supplementary Material.

#### Source reconstruction at 250–300 ms time window

**Fig. 1:**
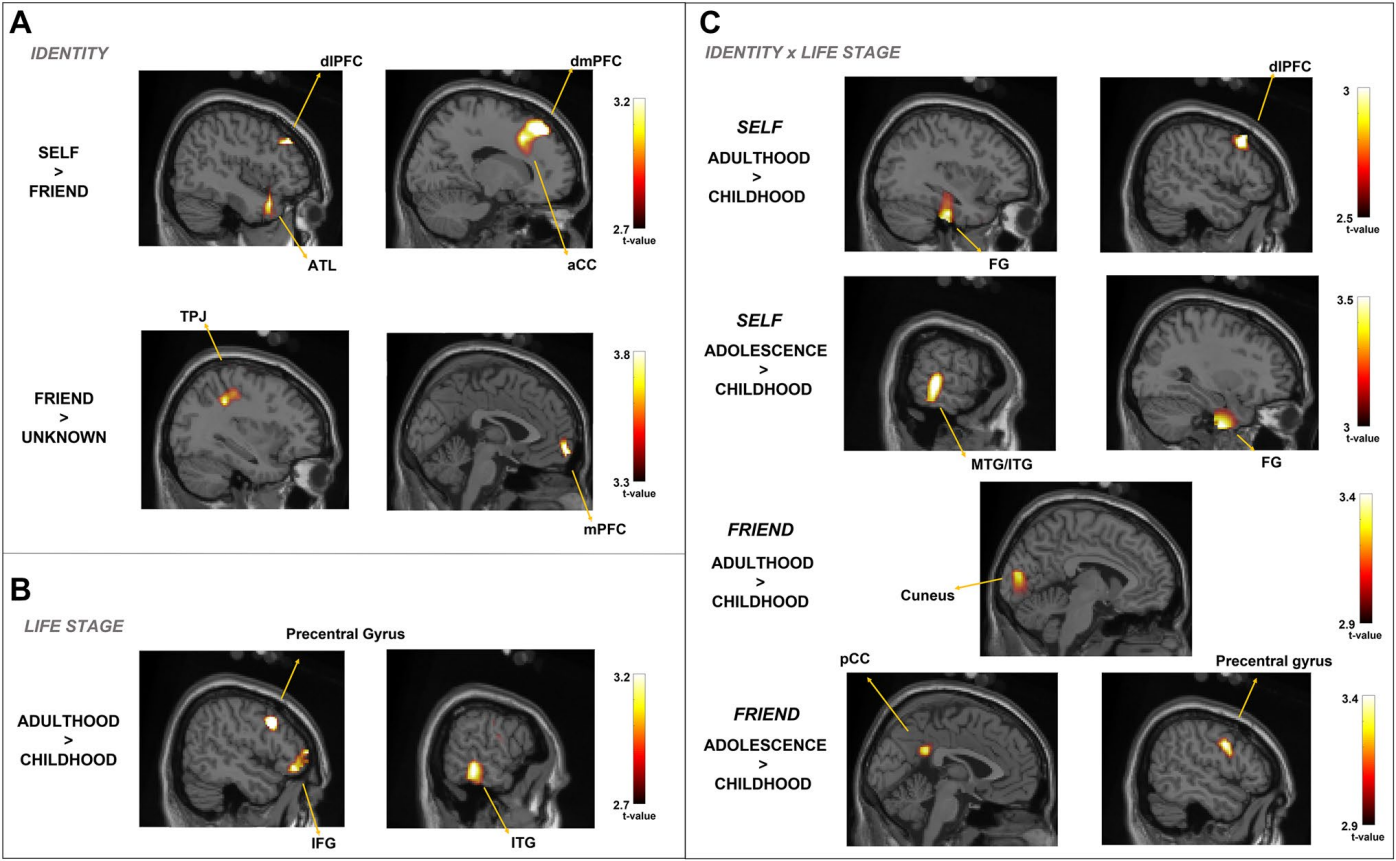
Source plots of the statistical t-maps or univariate contrasts corresponding to Identity effects (**A**), Life Stage (**B**) and interaction Identity × Life Stage (**C**) during the 250–300 ms time window. Reported brain regions significantly activated at a posterior statistical threshold *p* < 0.01. *aCC* anterior cingulate cortex, *ATL* anterior temporal lobule, *dlPFC* dorsolateral prefrontal cortex, *dmPFC* dorsomedial prefrontal cortex, *FG* fusiform gyrus, *IFG* inferior frontal gyrus, *ITG* inferior temporal gyrus, *MTG* middle temporal gyrus, *pCC* posterior cingulate cortex, *TPJ* temporoparietal junction

The cluster-permutation test indicated a significant effect for the contrasts of Self-identity vs. Close friend (*p* = 0.036), showing activated voxels in the dl/dmPFC, ATL and ACC; and Self-identity vs. Unknown (*p* = 0.008), in the vmPFC and TPJ. No significant effect was found for the contrast Close-friend vs. Unknown. Table 1 shows the MNI coordinates associated with the peak *t*-value for each significant contrast. Figure 1 shows the voxels that exceed the statistical threshold and corresponds to the peak t-value during this time window. Moreover, the cluster-permutation test for the Life Stage factor also yielded significant differences involving the contrast Adulthood vs. Childhood (*p* = 0.004), reflecting activated voxels in the precentral gyrus, IFG (pars opercularis), and Inferior Temporal Gyrus.

**Table 1:** Source reconstruction results during the 250–300 ms time window

Contrasts	Region-(hemisphere)	MNI coordinates			Peak *t*-values
		*x*	*y*	*z*	
Identity					
Self > Friend	Dorsomedial prefrontal cortex-R	16	32	50	3.25
	Dorsolateral prefrontal cortex-R	42	36	34	3.13
	Anterior cingulate cortex-R	16	18	40	3.02
	Anterior temporal lobule-R	44	16	− 30	3.01
	Dorsolateral prefrontal cortex-L	− 32	46	34	2.98
Self > Unknown	Medial prefrontal cortex-L	− 2	62	− 10	3.79
	Temporo-parietal junction-L	− 34	− 42	38	3.66
Life stage					
Adulthood > Childhood	Precentral gyrus-R	50	12	34	3.25
	Inferior frontal gyrus-R (pars opercularis)	52	30	− 6	2.85
	Posterior inferior temporal gyrus-L	− 60	− 34	− 16	3.03
Identity × life stage					
SelfAdult > SelfChild	Dorsolateral prefrontal cortex-L	50	18	42	3.05
	Fusiform gyrus-R	24	− 7	− 44	2.92
	Fusiform gyrus-L	− 36	− 20	− 32	2.88
SelfAdol > SelfChild	Middle temporal gyrus-R	64	− 34	− 4	3.59
	Inferior temporal gyrus-R	66	− 36	− 18	3.50
	Fusiform gyrus-R	30	− 8	− 36	3.35
FriendAdult > FriendChild	Cuneus-L	− 6	− 86	10	3.41
FriendAdol > FriendChild	Precentral gyrus-R	52	2	34	3.46
	Posterior cingulate cortex-L	− 2	− 46	28	3.03

Reported brain regions correspond to the MNI coordinates and significant peak *t*-values at *p* < 0.01. *Adol* adolescence, *Adult* adulthood, *Child* childhood, *MNI* Montreal National Institute

Concerning the interaction between Identity vs. Life Stage, the cluster-permutation test yielded significant differences for the contrast Adulthood vs. Childhood only for self-identity (*p* = 0.002), observing voxels activated in the dlPFC and fusiform gyrus, as well as for the contrast Adolescence vs. Childhood (*p* = 0.027) in the middle and inferior temporal cortices. Moreover, the contrasts between Adult vs. Infant and Adolescence vs. Childhood also yielded significant differences only for close-friend (*p* = 0.044 and *p* = 0.033, respectively), observing activated voxels in the cuneus, PCC, and precentral gyrus.

#### Source reconstruction at 300–600 ms time window

**Fig. 2:**
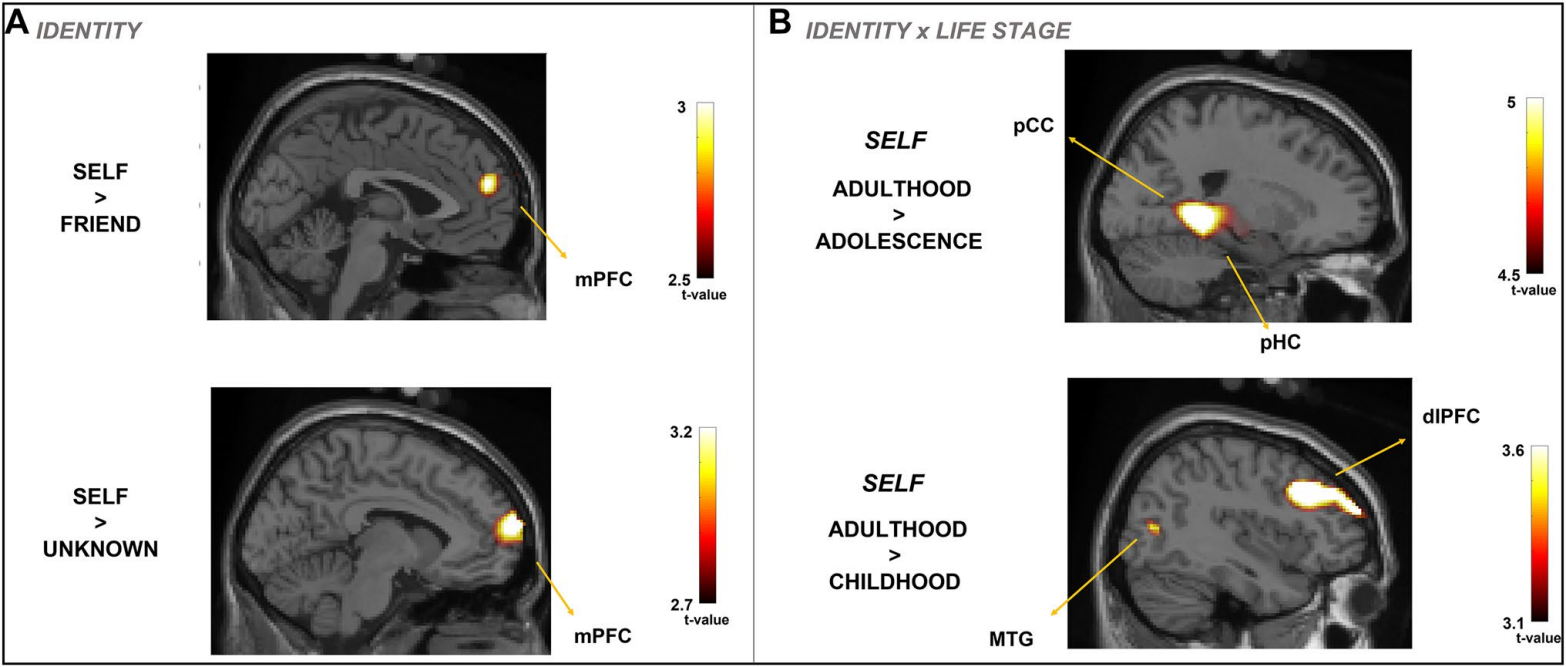
Source plots of the statistical t-maps for univariate contrasts corresponding to Identity effects (**A**) and the interaction Identity × Life Stage (**B**) during the 300–600 ms time window. Reported brain regions significantly activated at a posterior statistical threshold *p* < 0.01. *dlPFC* dorsolateral prefrontal cortex, *FG* fusiform gyrus, *IFG* inferior frontal gyrus, *mPFC* medial prefrontal cortex, *MTG* middle temporal gyrus, *pHC* parahippocampal cortex, *pCC* posterior cingulate cortex

The cluster-permutation test yields significant effects for the contrasts between Self-identity vs. Close-friend (*p* = 0.026), and Self-identity vs. Unknown (*p* = 0.017), showing activated voxels in the mPFC. The contrast Close-friend vs. Unknown did not yield significant statistical effects. Table 2 shows the MNI coordinates associated with the peak t-value for each significant contrast, and Fig. 2 shows those voxels that survive the threshold during this time window.

**Table 2:** Main significant results during the 300–600 ms time window

Contrasts	Region-(hemisphere)	MNI coordinates			Peak *t*-values
		*x*	*y*	*z*	
Identity					
Self > Friend	Medial prefrontal cortex-L	− 2	52	20	2.89
Self > Unknown	Medial prefrontal cortex-L	− 8	66	14	3.25
Identity × life stage					
SelfAdu > SelfAdo	Precuneus/posterior cingulate cortex-R	21	− 44	0	5.32
	Parahippocampal gyrus-R	20	− 38	− 6	5.08
	Fusiform gyrus-R	28	− 40	− 12	4.21
SelfAdu > SelfInf	Dorsolateral prefrontal cortex-R	40	20	34	3.72
	Middle temporal gyrus-R	42	74	8	3.48

*Adol* adolescence, *Adult* adulthood, *Child* childhood, *MNI* Montreal National Institute

Concerning the Identity vs. Life Stage interaction, the cluster-permutation test yielded significant effects for the contrasts between Adulthood vs. Adolescence and Adulthood vs. Childhood only for self-identity condition (*p* = 0.002 and *p* = 0.038, respectively), reflecting activations of posterior (PC/PCC) temporal (FG, MTG, paraHC) and anterior regions (dlPFC). The contrast between Adolescence vs. Childhood for the Self-Identity condition was non-significant (*p* = 0.7).

## Discussion

This study aimed to disentangle the spatio-temporal pattern of the estimated brain sources power, relative to diachronic (recognition of self vs. others faces regardless of time passing) and synchronic (recognition of the self at different life stages) components of self-identity. Briefly, no global face configuration processing in the 150–200 ms time window was found. Later (250–300 ms), an increment in source power were found in dm/dlPFC, ACC and ATL differentiating between self-identity and close-familiar. The contrast between self-identity vs. unknown showed an increment of power in the mPFC and TPJ. Moreover, the interaction between Life Stage and Identity showed a distributed net of areas specific to self-identity, contrasting adulthood vs. childhood encompassing dlPFC and FG, and adolescence vs. childhood (middle and inferior temporal cortices). Finally, the 300–600 ms time window showed specific effects in the source power in the mPFC, distinguishing self-identity vs. close-familiar and self-identity vs. unknown. Regarding Life stage, only self-identity in adulthood vs. adolescence yielded significant clusters in PCC and paraHC, and adulthood vs. childhood in dlPFC and MTG. These results will be discussed in detail in the following paragraphs.

### Facial familiarity effects depend on the degree of self-relevance

Contrary to our prediction, the cluster-permutation test was not sensitive to revealing any significant cluster in this early time window. By contrast, significant effects related to self-identity were found in the 250–300 ms time windows. The pattern of source power was somewhat different in the contrast self-identity vs. close-friend corresponding to the clusters dm- and dl-PFC, ATL, and ACC, compared to self-identity vs. unknown corresponding to the clusters mPFC and TPJ. Such dissimilar patterns of brain sources may reflect a differential processing in self-face recognition from personal knowledge depending on the degree of relationship to self. Moreover, this pattern of results encompasses those found in our ERP study related to N250 modulations, specifically to self-faces (Rubianes et al. 2020). Other authors have linked this component to face familiarity as indexing the access to long-term memory (Olivares et al. 2015) or accessing semantic information associated with that familiar face (Paller et al. 2000). This extended system was largely activated for self-identity, conveying more autobiographical information to discriminate against other face identities, even related to the self (close-familiar). Recognizing self vs. close-friend recruits executive areas in the midline (dmPFC and ACC) and lateral cortices (dlPFC and ATL), which may reveal more effort to discriminate self from close friend than from unknown based on extensive person knowledge retrieval (Bobes et al. 2018). Arguably, when recognizing self-identity from other identities, prioritized processing by medial areas is regulated by lateral areas, probably by inhibitory connectivity (Humphreys and Sui 2016). This claim is substantiated by studies that evinced medial cortices, most allowing to the default mode network are modulated by executive cortices (dlPFC) in the continuous personal-relevance to non-personal-relevance (Sui and Gu 2017; Sui and Rotshtein 2019). In fact, TPJ and ATL, belonging to the DMN (Shulman et al. 1997), are engaged in internal attention orientation when discriminating self-related vs. other-related information (Fox et al. 2005). In addition, mPFC, ATL and ACC are anatomically interconnected in higher cognitive functions, namely, self-processing and social cognition (Knyazev et al. 2018). Several studies have found a gradient ventral-to-dorsal PFC in self-processing, being the vmPFC more specific to self and dmPFC is to other (Denny et al. 2012; Wagner et al. 2013). Our results showed that both are involved in self vs. other discrimination, but in different contrasts, namely, vmPFC to self vs. unknown and dmPFC to self vs. close-friend. Since vm/dmPFC constitute regions recruited in self-referential processes (Northoff and Bermpohl 2004), in particular, introspection and emotional reaction (Roy et al. 2012; Rolls et al. 2019), the contrast self-identity vs. close-friend and self-identity vs. unknown involved different emotional attribution and reward value.

Concerning the Identity vs. Life Stage interaction, we observed a specific effect of self-identity at different life stages entailing a frontotemporal network in the core face areas (FG), and executive areas (dlPFC). While self-identity in adulthood vs. childhood entails frontotemporal areas (dlPFC and FG), self-identity in adolescence vs. childhood entails only temporal areas (MTG, FG). Therefore, self-identity in adulthood seems to recruit a distributed net of areas than self at distant life stages (adolescence and childhood). Though significant, close-friend processing in adulthood and childhood elicited a restricted extension of areas relative to early visual processing stages (cuneus and PCC). Murray et al. (2015) observed that the mPFC, FG, and other visual areas maintain self-vs-other memory-matched perceptual representation in the socially oriented perspective. Moreover, D’Argembeau et al. (2008, 2010) suggested that access to autobiographical memory is supported by a frontal-posterior link, entailing face-specific areas and memory-specific areas. Such areas are boosted when face identities are relevant to participants, even when they are unaware of it.

In conclusion, compared to self-identity recognition, close-familiar faces summoned more attentional resources accessing autobiographical memory than unknown faces that are treated more holistically (Buttle and Raymond 2003; Farah et al. 1995). Moreover, we found a significant effect in source power in dlPFC and fusiform comparing self-identity to adulthood and childhood as an index of top-down executive processing relative to keeping an updated representation of self-identity.

### Anterior–posterior areas underlie the unity of self-awareness in lifespan

Later latencies (300–600 ms), the mPFC appeared more specific discriminating self vs. close-friend and unknown faces irrespective of life stages. In addition, self-identity in adulthood showed higher activation in PCC and paraHC than in adolescence, and the dlPFC and MTG when comparing self in adulthood to childhood. Murray et al. (2015) pointed out that posterior areas may distinguish self from non-self by implementing a functional self- vs. non-self face distinction in perception, cognition and emotion (Conway et al. 2004).

This finding endorses the hypothesis that mPFC seems to participate in discriminating self vs. close-friend and unknown identities based on differential autobiographical contents, engaging deeper attribution of reward value and personal meaning (Conway et al. 2004; Gobbini and Haxby 2007; Murray et al. 2015; Tanguay et al. 2018). In Rubianes et al. (2020), we observed specific self-relevant processing in this time window indexed by the P3-LPC. It is likely that the mPFC, PCC and paraHC comprise neural sources of the P3-LPC components as key multimodal areas of accessing autobiographical memory of meaningful self-related information (see also Muñoz et al. 2020 using an ownership paradigm). Regarding life stages, the paraHC and PCC also exhibited a self-prioritized effect in adulthood compared to adolescence, which may be involved in top-down attentional processes (Kim et al. 2020). Although mPFC was not observed in the interaction Identity vs. Life stage, the mPFC might be involved in self-identity processing at different life stages together with paraHC and PCC. This claim is supported by the evident activation of the mPFC only in self-identity but not in close-friend and unknown faces. We suggest that the mPFC, together with dlPFC may exert some top-down facilitation over posterior areas, enhancing the processing of self-facial configuration as a likely role in reallocating processing resources for socially rewarding information, while the PCC, paraHC, MTG region is representing self-relevant information across time (Góngora et al. 2019).

As we mentioned, the mPFC and the PCC, paraHC showed self-specificity mainly at present ages (adulthood). The fact that no specificity was found to other face identities (close-friend and unknown) leads us to surmise that later stages entail the access and holding of an updated representation of the identity of the self, as feasible evidence of the diachronic component. However, our findings also indicate that the PCC and paraHC convey the temporal perspective to identities in the life stages task. Not only higher activity in the mPFC and dlPFC was specific to self-identity recognition at present, but also the PCC and MTG were differentially sensitive to adolescence and childhood, which was not observed in other identities. In sum, while mPFC pointed to a diachronic component, the PCC, MTG, and paraHC discriminated self-identity in the present from adolescence and childhood, compatible with the synchronic component. Several authors observed that the mPFC supports self-referential compared to other-referential information, though higher activity in this area has been observed when referring to the judgment of facial self (D’Argembeau et al. 2008, 2010; Northoff 2017; Sugiura et al. 2012). The present work extends this observation by adding the PCC, paraHC, and MTG, just recognizing one’s current, recent and distant past facial appearance.

Some limitations of this study must be addressed. Firstly, the first limitation of this study is the restricted sample size, which may be limiting the statistical power. It was challenging to collect participants that should provide us with the required set of high-quality photos (their faces and a close friend’s faces at different ages). Moreover, unlike parametric statistics, bootstrap statistics is distribution-independent, so that it may account for not fully representative samples. The second limitation concerns the analysis of the same stimulus presented twice (identity recognition and life stages recognition tasks) to enhance the signal-to-noise ratio and facilitate statistical analysis. Thus, it is likely that identity processes (e.g., self-relevance) were taking place implicitly during the life stages recognition task, while life stage processes (e.g., recognition of children’s faces) were taking place implicitly during the identity recognition task. Cluster-permutation tests collapsed both explicit and implicit, therefore these processes have not biased the results.

## Conclusions

Brain correlates of self-identity encompass anterior and posterior areas intertwining bottom-up and top-down processes across the neural time course. At middle latencies, dl/dmPFC and ACC dynamics, together with ATL, indexed the access to familiar faces depending on self-related contents (self-identity vs. close friend). Self-identity vs. unknown recognition entails mPFC and TPJ likely indexing to take another perspective. Later processes were concerned with discriminating self-specific from other identities, involving the mPFC. While mPFC maintained an updated representation of self-identity (diachronic self) based on current rewarding value, the dlPFC and FG, MTG, paraHC, PCC were sensitive to different life stages of self-identity (synchronic self) during the access to autobiographical memory.

## Supplementary Information

Below is the link to the electronic supplementary material.

Supplementary file1 (DOCX 56 KB)

## Data Availability

The data used for the statistical analyses are available in the Open Science Framework (OSF) online repository (https://osf.io/9hfd5/). See supplementary material for a full report of all statistical comparisons.
