# Supplementary material for: Spatio-temporal brain dynamics of self-identity: an EEG source analysis of the current and past self
Source: Brain Struct Funct. 2022 Jun 7;227(6):2167–79. doi: 10.1007/s00429-022-02515-9 (PMC9232421; doi:10.1007/s00429-022-02515-9)
Supplement: Supplementary file 1 — Supplementary file1 (DOCX 56 KB) [file 429_2022_2515_MOESM1_ESM.docx]

# SUPPLEMENTARY INFORMATION

**DESCRIPTION OF THE RESULTS**

**Power source at 150-200 ms time-window**

| **Contrasts** | **Cluster p-value** |
| --- | --- |
| ***Identity*** |  |
| Self > Friend | 0.7138 |
| Self > Unknown | 0.8376 |
| Friend > Unknown | 0.2700 |
| ***Life Stage*** |  |
| Adulthood > Adolescence | 0.2862 |
| Adulthood > Childhood | 0.5573 |
| Adolescence > Childhood | 0.6129 |
| ***Identity x Life Stage*** |  |
| Self (Adulthood > Adolescence) | 0.1756 |
| Self (Adulthood > Childhood) | 0.5057 |
| Self (Adolescence > Childhood) | 0.6363 |
| Friend (Adulthood > Adolescence) | 0.4061 |
| Friend (Adulthood > Childhood) | 0.5091 |
| Friend (Adolescence > Childhood) | 0.5767 |
| Unknown (Adulthood > Adolescence) | 0.3607 |
| Unknown (Adulthood > Childhood) | 0.4717 |
| Unknown (Adolescence > Childhood) | 0.6025 |

**Power source at 250-300 ms time-window.**

| **Contrasts** | **Cluster p-value** |
| --- | --- |
| ***Identity*** |  |
| **Self > Friend** | **0.036** |
| **Self > Unknown** | **0.0079** |
| Friend > Unknown | 0.7037 |
| ***Life Stage*** |  |
| Adulthood > Adolescence | 0.1290 |
| **Adulthood > Childhood** | **0.0411** |
| Adolescence > Childhood | 0.0877 |
| ***Identity x Life Stage*** |  |
| Self (Adulthood > Adolescence) | 0.4573 |
| Self (Adulthood > Childhood) | **0.0184** |
| Self (Adolescence > Childhood) | **0.0270** |
| Friend (Adulthood > Adolescence) | 0.5227 |
| Friend (Adulthood > Childhood) | **0.0437** |
| Friend (Adolescence > Childhood) | **0.0326** |
| Unknown (Adulthood > Adolescence) | 0.3131 |
| Unknown (Adulthood > Childhood) | 0.7291 |
| Unknown (Adolescence > Childhood) | 0.8482 |

**Power source at 300-600 ms time-window**

| **Contrasts** | **Cluster p-value** |
| --- | --- |
| ***Identity*** |  |
| **Self > Friend** | **0.0262** |
| **Self > Unknown** | **0.0166** |
| Friend > Unknown | 0.5319 |
| ***Life Stage*** |  |
| Adulthood > Adolescence | 0.0788 |
| Adulthood > Childhood | 0.1702 |
| Adolescence > Childhood | 0.3171 |
| ***Identity x Life Stage*** |  |
| **Self (Adulthood > Adolescence)** | **0.0014** |
| **Self (Adulthood > Childhood)** | **0.0384** |
| Self (Adolescence > Childhood) | 0.7067 |
| Friend (Adulthood > Adolescence) | 0.5295 |
| Friend (Adulthood > Childhood) | 0.4441 |
| Friend (Adolescence > Childhood) | 0.3509 |
| Unknown (Adulthood > Adolescence) | 0.4401 |
| Unknown (Adulthood > Childhood) | 0.9172 |
| Unknown (Adolescence > Childhood) | 0.9756 |
